# Supplementary material for: Assessing the role of vascular risk factors in dementia: Mendelian randomization meta-analysis and comparison with observational estimates
Source: F1000Res. 2025 Feb 7;11:565. Originally published 2022 May 24. [Version 2] doi: 10.12688/f1000research.121604.2 (PMC11880757; doi:10.12688/f1000research.121604.2)
Supplement: Peer review history

# Reviewer report: Reviewer response for version 2

**DOI:** 10.5256/f1000research.177829.r365155

**Published:** 2025-03-04

**Authors:** Ahmet Turan Isik

**Affiliations:** 1 Dokuz Eylul University, İzmir, Turkey

I would like to thank the authors for revising the MN according to the recommendations. I believe it is now ready to be indexed.

Are the rationale for, and objectives of, the Systematic Review clearly stated?

No

Is the statistical analysis and its interpretation appropriate?

I cannot comment. A qualified statistician is required.

If this is a Living Systematic Review, is the ‘living’ method appropriate and is the search schedule clearly defined and justified? (‘Living Systematic Review’ or a variation of this term should be included in the title.)

Not applicable

Are sufficient details of the methods and analysis provided to allow replication by others?

Yes

Are the conclusions drawn adequately supported by the results presented in the review?

Yes

Reviewer Expertise:

Cognitive Impairement in older adults, NPH, Dysautonomia in DLB

I confirm that I have read this submission and believe that I have an appropriate level of expertise to confirm that it is of an acceptable scientific standard.

# Reviewer report: Reviewer response for version 1

**DOI:** 10.5256/f1000research.133485.r307125

**Published:** 2024-09-27

**Authors:** Ahmet Turan Isik

**Affiliations:** 1 Dokuz Eylul University, İzmir, Turkey

I have reviewed the manuscript "Assessing the role of vascular risk factors in dementia: Mendelian randomization meta-analysis and comparison with observational estimates" with interest. The paper needs to reconsider a few flaws.

 The background and purpose of the study should be emphasized more clearly.

 The authors should explain the effects of any potential overlap between the cohorts on the results.

 It would be better for a statistician to recheck the results because I am not good enough at this topic.

**No competing interests were disclosed**

Are the rationale for, and objectives of, the Systematic Review clearly stated?

No

Is the statistical analysis and its interpretation appropriate?

I cannot comment. A qualified statistician is required.

If this is a Living Systematic Review, is the ‘living’ method appropriate and is the search schedule clearly defined and justified? (‘Living Systematic Review’ or a variation of this term should be included in the title.)

Not applicable

Are sufficient details of the methods and analysis provided to allow replication by others?

Yes

Are the conclusions drawn adequately supported by the results presented in the review?

Yes

Reviewer Expertise:

Cognitive Impairement in older adults, NPH, Dysautonomia in DLB

I confirm that I have read this submission and believe that I have an appropriate level of expertise to confirm that it is of an acceptable scientific standard, however I have significant reservations, as outlined above.

# Response

**Published:** 2025-02-03

**Authors:** Liam Lee

**Affiliations:** University of Edinburgh, UK

Dear Dr Isik, 

 Many thanks for your valuable feedback on our manuscript. 

 We have carefully considered your comments and made the following changes to address them.

**Comment 1: The background and purpose of the study should be emphasized more clearly.**

 We have revised the background section to provide a clearer explanation of the rationale and purpose of the study. This includes highlighting the limitations of observational studies in inferring causality and the importance of using Mendelian randomization (MR) as a complementary approach.

**Comment 2: The authors should explain the effects of any potential overlap between the cohorts on the results.**

 We have added a discussion on the effects of cohort overlap in the **Limitations** section of the manuscript. We acknowledge that cohort overlap could lead to bias, such as inflated precision and potential under- or overestimation of effect sizes. To address this, we emphasized our sensitivity analyses by substituting alternative estimates where possible and found that the overall pooled estimates remained stable. 

**Comment 3: It would be better for a statistician to recheck the results because I am not good enough at this topic.**

 We would like to reassure the reviewer that one of the co-authors, Dr. Walker, is a trained epidemiologist with extensive experience in statistical methods and MR studies. Dr. Walker has validated the statistical analyses and interpretation presented in the manuscript.

# Reviewer report: Reviewer response for version 1

**DOI:** 10.5256/f1000research.133485.r157914

**Published:** 2023-01-19

**Authors:** Daniel A Nation

**Affiliations:** 1 University of California, California, USA

This is a meta-analysis of mendelian randomization studies and a comparison of this meta-analysis with observational study estimates, to evaluate the potential causal nature of observed associations between 9 vascular risk factors and dementia. The authors conducted a thorough search and identified 12 out of 5211 studies meeting criteria for inclusion in the analysis. Findings indicated higher genetically predicted BMI is associated with slightly higher odds of dementia. Furthermore, fewer vascular risk factors were associated with dementia in mendelian randomization studies than in observational studies. The question of a causal role for modifiable vascular risk factors for dementia is critical to efforts to treat and prevent cognitive impairment, making the topic of this article particularly important. Strengths of the present study include examination of MR which may allow for causal inference, meta-analysis of MR studies in this area which is novel, and comparison with observational studies. The methods appear to be rigorous. Unfortunately, very little can be concluded from the study due to limitations of available data within MR studies themselves and across studies. Although the authors extensively outline these limitations in excellent detail, the limits curtail the ultimate impact of this study. Nevertheless, it was well written and perhaps serves as an initial step forward or a snapshot of field as it currently stands, including the many remaining questions.

Are the rationale for, and objectives of, the Systematic Review clearly stated?

Yes

Is the statistical analysis and its interpretation appropriate?

I cannot comment. A qualified statistician is required.

Are sufficient details of the methods and analysis provided to allow replication by others?

Yes

Are the conclusions drawn adequately supported by the results presented in the review?

Yes

Reviewer Expertise:

Vascular factors contributing to dementia

I confirm that I have read this submission and believe that I have an appropriate level of expertise to confirm that it is of an acceptable scientific standard.
